# Network pharmacology: a bright guiding light on the way to explore the personalized precise medication of traditional Chinese medicine

**DOI:** 10.1186/s13020-023-00853-2

**Published:** 2023-11-08

**Authors:** Ling Li, Lele Yang, Liuqing Yang, Chunrong He, Yuxin He, Liping Chen, Qin Dong, Huaiying Zhang, Shiyun Chen, Peng Li

**Affiliations:** 1 School of Comprehensive Health Management, Xihua University, Chengdu, Sichuan China; 2 Chengdu University of Traditional Chinese Medicine, Chengdu, Sichuan China; 3 State Key Laboratory of Quality Research in Chinese Medicine, Institute of Chinese Medical Sciences, University of Macau, Macau, China; 4 School of Food and Bioengineering, Xihua University, Chengdu, Sichuan China; 5 Zhuhai UM Science and Technology Research Institute, Zhuhai, Guangdong China

**Keywords:** Traditional Chinese medicine, Network pharmacology, Research strategy, Precision treatment, Application

## Abstract

Network pharmacology can ascertain the therapeutic mechanism of drugs for treating diseases at the level of biological targets and pathways. The effective mechanism study of traditional Chinese medicine (TCM) characterized by multi-component, multi-targeted, and integrative efficacy, perfectly corresponds to the application of network pharmacology. Currently, network pharmacology has been widely utilized to clarify the mechanism of the physiological activity of TCM. In this review, we comprehensively summarize the application of network pharmacology in TCM to reveal its potential of verifying the phenotype and underlying causes of diseases, realizing the personalized and accurate application of TCM. We searched the literature using “TCM network pharmacology” and “network pharmacology” as keywords from Web of Science, PubMed, Google Scholar, as well as Chinese National Knowledge Infrastructure in the last decade. The origins, development, and application of network pharmacology are closely correlated with the study of TCM which has been applied in China for thousands of years. Network pharmacology and TCM have the same core idea and promote each other. A well-defined research strategy for network pharmacology has been utilized in several aspects of TCM research, including the elucidation of the biological basis of diseases and syndromes, the prediction of TCM targets, the screening of TCM active compounds, and the decipherment of mechanisms of TCM in treating diseases. However, several factors limit its application, such as the selection of databases and algorithms, the unstable quality of the research results, and the lack of standardization. This review aims to provide references and ideas for the research of TCM and to encourage the personalized and precise use of Chinese medicine.

## Introduction

The “one drug–one target–one disease” model is widely recognized as the paradigm in developing new medicines, which simplifies the screening of compounds and reduces the unwanted side effects of medication [1–3]. Highly selective therapeutic drugs against a single target have been shown to have limited efficacy and therapeutic effects, particularly for complex multifactorial diseases whose pathogenesis are modulated by diverse biological processes and various molecular functions [4]. It is obvious that the path of modulating multiple biological processes by designing highly selective compounds alone is not viable. In fact, in the past few decades, a constant reduction in the overall success rate of the development of clinical intervention agents has been observed [5]. Due to the complex signaling networks of diseases, multi-target and combinatorial drug therapy provide a new network-based approach to drug discovery [6]. Networks not only improve the therapeutic efficacy of drugs while predicting unwanted side effects but also provide a broader choice of disease targets, which revolutionizes the definition and treatment of diseases. With an increased understanding of the underlying therapeutic mechanisms of approved drugs on the market, it is demonstrated that many drugs with definite efficacy do not act on only one target, but frequently on multiple targets, such as anti-epileptic drugs Felbamate and Topiramate [7]. In addition, taking advantage of independent action targets and complementary mechanisms of action with more therapeutic benefit and less toxicity and resistance, the combination treatment is superior to monotherapy [8–11], as shown in the fixed combination of Vildagliptin/Metformin in type 2 diabetic patients [12]. The network pharmacology paradigm provides a bioinformatics network that demonstrates multiple disease genes and drug target genes are interconnected, thereby illustrating the drug-disease interactions and guiding the development and application of innovative drugs.

Meanwhile, the development of traditional Chinese medicine (TCM) has long been challenged and shackled by the modern “one drug–one target–one disease” dogma. Due to the complexity of compounds in TCM and the multi-targeted mechanism, the principle of drug development of TCM is moving away from clarifying holistic theory towards screening highly selective compounds from herbal medicines. However, this approach does not take into account the holistic and integrative efficacy of herbs. It is estimated that approximately one-third of approved drugs are derived from natural products and their derivatives [13]. The screened active compounds cannot reveal the integrative efficacy of herbal medicines and control their quality. Fortunately, in the form of compound preparations, TCM has been recommended and approved for effectively preventing and treating COVID-19 in China [14], and this natural model of combination therapy can revolutionize drug development. The rich TCM theory may facilitate the decipherment of the molecular mechanisms of drug combinations, enabling the clinical intervention of diseases according to their manifestation and root cause. Network pharmacology provides the possibility for the personalized precise medication of TCM by deciphering its efficacy through the scientific method. The review introduces the initial origin and subsequent development of network pharmacology, as well as its applications in the research fields of the precision treatment of TCM, to hopefully provide ideas and references for the personalized and precise use of traditional Chinese medicine.

## The development history of network pharmacology

**Fig. 1:**
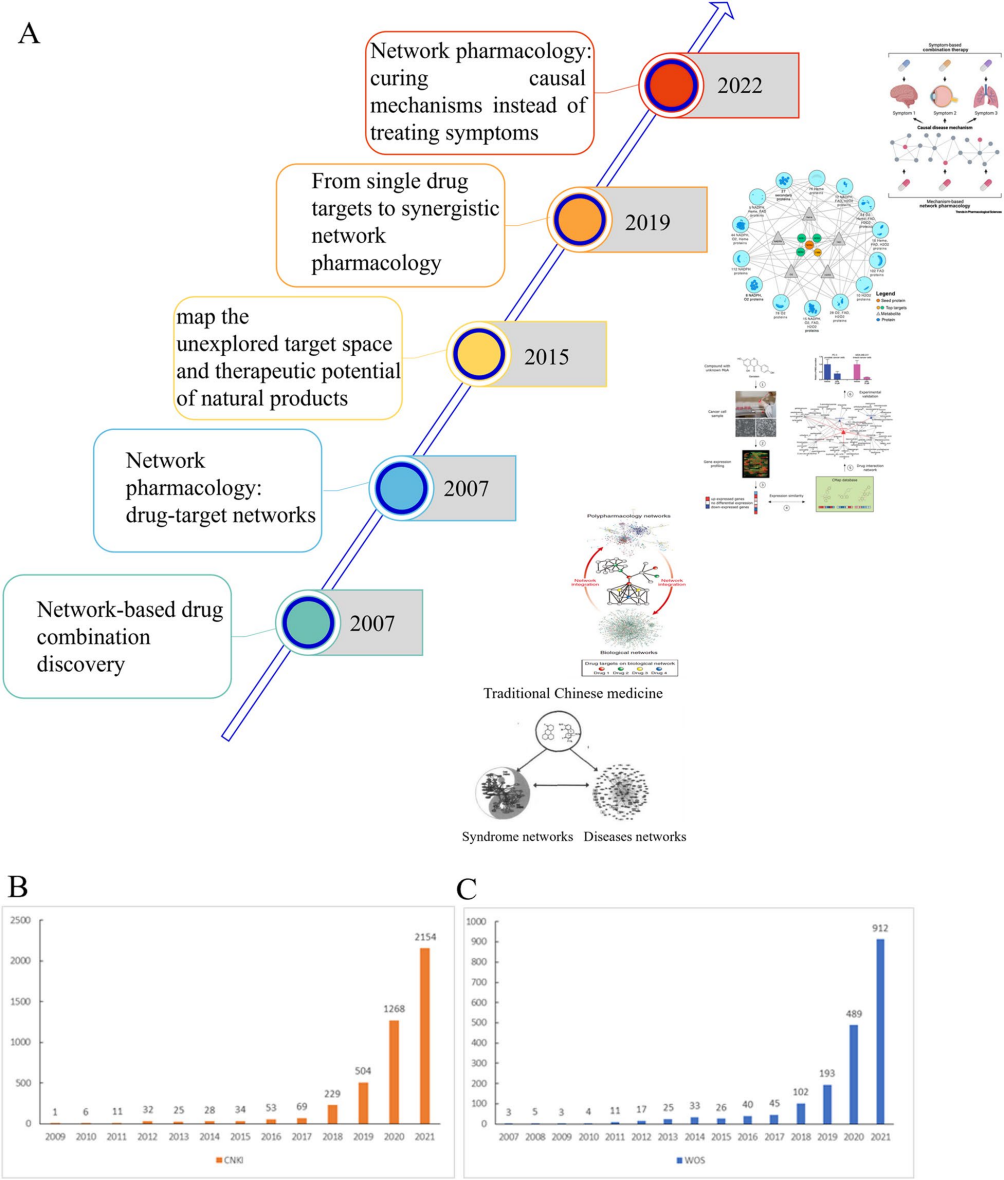
The timeline of major milestones for the development history of network pharmacology **A**. The number of papers published in the field of network pharmacology in CNKI **B** and WOS **C**. Reproduced from Ref [1, 3, 17, 23, 24]

The origin of network pharmacology can be traced back to 1999 when Shao Li pioneered the “Syndrome” of a link between TCM and biomolecular networks at the first annual academic conference of the Chinese Association for Science and Technology [15]. A few years later, he suggested that the disease gene network might be regulated by the “multi-causal and micro-effective” effects of herbal formulae [16]. In 2007, Li et al. used bioinformatics to construct the first biomolecular network of Cold /Hot syndrome in TCM and found the network regulatory effects of the formulae for Cold /Hot syndrome [17]. In the same year, “Network Pharmacology” was introduced by Andrew L. Hopkins, a pharmacologist at Dundee University in the UK [1]. Figure 1A shows the development history of network pharmacology. Subsequently, network pharmacology has increasingly become a hot topic in pharmaceutical research. The search of the Chinese National Knowledge Infrastructure (CNKI) databases and Web of Science (WOS) showed that the number of articles about network pharmacology has increased dramatically in recent years (Fig. 1B, C). In particular, it has gained momentum in recent years and is expected to become a promising paradigm for the new generation of drug development [18]. In 2009, Pan Jiahu established a new model for drug discovery by using network pharmacology [19]. More recently, it has become a popular research topic in systematic pharmacological research, especially in the field of research on the pharmacodynamic mechanism of TCM, due to the high degree of overlap between the main ideas of network pharmacology and those of TCM. Next, Li established a “phenotypic network-biological network-Chinese medicine network” model for TCM evidence and TCM prescription research in the same year [20]. He proposed the idea of “network targets” for the first time two years later [21] and created a collaborative algorithm for predicting drug combinations through network targets. In 2021, to increase the credibility of results and standardize the feasibility of data, Li’s team developed and published the first international standard for network pharmacology “Guidelines for Evaluation Methods in Network Pharmacology” [22].

## Network pharmacology bumps into TCM

The origins and development of network pharmacology are tightly correlated with the study of TCM. Network pharmacology is a unique system established on the medical knowledge of traditional experience [25] and is fundamentally different from the “drug-target-disease” theoretical system of modern medicine [26]. TCM experience emphasizes the ideas of “diagnosis and treatment” and “holistic view” in the treatment of diseases, providing a natural model to study combination therapy. Specifically, Chinese herbs are usually in compounded preparations to form TCM formulas following the TCM principles of “*the seven methods in prescription compatibility*” and “*Jun Chen Zuo Shi*” as the combined therapy. In recent years, TCM has gradually stepped into the international arena, and the modernization of TCM has become the core issue of TCM research, which needs scientific validity. However, because of the complex of TCM ingredients and their complicated interactions with biological systems, the molecular mechanisms of TCM components are difficult to elucidate. Revealing the molecular mechanism and bioactive markers of TCM, as well as its toxic mechanism are urgent problems to be solved in the standardization of TCM [27]. TCM treatment is a multi-compound, multi-targeted, and integrative paradigm, while network pharmacology is a systematic biology method for the analysis of multi-target and multi-pathway pharmacological effects. Thus, network pharmacology has become a common strategy for investigating the therapeutic mechanisms of TCM, which is proved useful for deciphering the scientific basis of TCM. Through network pharmacology approach, some good preliminary achievement have been obtained in exploring the essential characteristics of TCM, discovering the multiple effects of TCM with multiple pathways, multiple targets, and multiple components [21, 27–29]. Collectively, network pharmacology and TCM have same core idea and promote each other.

## Research strategies and application of network pharmacology

According to previous studies [30–34], a comprehensive search of Web of Science, PubMed, Google Scholar, as well as Chinese National Knowledge Infrastructure in the last decade was performed in this study. Using specific keywords related to “TCM network pharmacology” or “network pharmacology”, relevant articles including in silico, in-vitro or animal experiments and review articles were identified. Inclusion criteria: these selected articles were assessed and categorized for information about the databases for network pharmacology, network visualization and analysis, and the application of network pharmacology in TCM including elucidating the biological basis of diseases and syndromes, predicting the targets of TCM, screening bioactive substances, deciphering mechanisms. Exclusion criteria: articles uncovering the current issue and those with incomplete information, letter to the editor, and short communications. The research strategies and current application of network pharmacology in the field of TCM are comprehensive overviewed, offering guidance for utilizing network pharmacology to uncover the modern scientific essence of TCM based on previous work [35–37].

### Research strategies of network pharmacology

We firstly presented the research steps involved in network pharmacology. The procedures of network pharmacology focus on the following steps: (1) mapping the disease phenotypic targets and the drug targets together in the biomolecular network; (2) establishing the mechanism of association between diseases and drugs; (3) analyzing the network to dissect the mechanism between network targets and the system regulation. Network targets are the key ideas of network pharmacology [38, 39], which assumes that disease phenotypes and drugs act on the same network, same pathway, or even the same target, thus affecting the balance of network targets and interfering with the phenotype at all levels. Therefore, we summarized network pharmacology-related databases and strategy for network visualization and analysis.

#### Databases for network pharmacology

The model of network pharmacology is constructed by the collection which is based on screening of information on bioactive components, target genes, and disease genes in various databases. Many scholars and experts have developed databases related to network pharmacology, which integrate relevant information in the field of medicine and provide a basis for research in network pharmacology [40]. These databases are divided into several categories, including herb/compound databases, disease databases, and gene/protein databases. Commonly used databases for network pharmacology research involve TCMSP (http://sm.nwsuaf.edu.cn/lsp/tcmsp.php) [41], HERB(http://herb.ac.cn/) [42], TCMBank(https://www.tcmbank.com/) [43], HIT(http://lifecenter.sgst.cn/hit/) [44], ETCM (http://www.nrc.ac.cn:9090/ETCM/) [45], TCMID (http: //www.megabionet.org/tcm-id/) [46], CMAUP(CMAUP—Collective Molecular Activities of Useful Plants (bidd.group)) [47], YaTCM(http://cadd.pharmacy.nankai.edu.cn/yatcm/home) [48], TCM database@Taiwan (http://tcm.cmu.edu.tw) [49], Pubchem (https://pubchem.ncbi.nlm.nih.gov) [50], STRING (https://string-db.org) [51], Uniprot (https: //www.uniprot.org/) [52], Drugbank (www.drugb-ank.ca) [53], OMIM (https://www.omim.org/) [54], GeneCards (www.genecards.org) [55], DisGeNET (https://www.disgenet.org/api) [56], TTD (https://idrblab.org/ttd/) [57], DAVID (https://david.ncifcrf.gov) [58], Metascape (http://metascape.org) [59], and KEGG (https://www.kegg.jp/) [60] (Table 1).

**Table 1:** Databases related to network pharmacology

Database/ software category	Name	Description
Herbs/Compound	TCMSP	A systematic pharmacology database for finding and screening compounds, targets in Herbs
	HERB	A high-throughput experiment and reference-guided database of traditional Chinese medicine
	TCMBank	It provides standardized information about traditional Chinese medicines, ingredients, and establishes six pairs of relationships
	HIT	Herbal Ingredients' Targets Database is a comprehensive and fully curated database to complement available resources on protein targets for FDA-approved drugs as well as the promising precursors
	ETCM	A comprehensive database of herbal ingredients, herbs and formulations, predictive target genes, and systematic analysis
	TCMID	A comprehensive database providing information and connecting TCM and modern life sciences
	CMAUP	Collective Molecular Activities of Useful Plants provides collective molecular activities of useful plants, human target proteins, Gene Ontology, KEGG pathways, and their relations to human diseases
	YaTCM	It provides comprehensive biologically relevant information on isolated TCM compounds, including prescriptions, herbs, ingredients, definite or putative protein targets, pathways, and diseases
	TCM database@Taiwan	Non-commercial TCM database for screening Chinese medicine compounds
	PubChem	A chemical database with information on small molecules as well as some large molecules
Gene/ Protein	STRING	A database focusing on protein–protein associations
	UniProt	A freely accessible protein sequence and annotation database
Disease	Drugbank	A comprehensive database of molecular information on drugs, mechanisms of action, interactions, and targets
	OMIM	A daily updated database of human genes and genetic phenotypes
	GeneCards	A comprehensive database providing annotation information on human genes
	DisGeNET	A comprehensive database providing relationships between genes and variants associated with human diseases
	TTD	A database to provide information about the targets, pathways, and disease together with the corresponding drugs directed at the targets
Functional annotations	DAVID	Database providing a web server for functional annotation, visualization, and integrated discovery of gene lists
	Metascape	A Database for gene annotation and analysis resource
	KEGG	Kyoto Encyclopedia of Genes and Genomes is a database resource for understanding high-level functions and utilities of the biological system

#### Network visualization and analysis

**Fig. 2:**
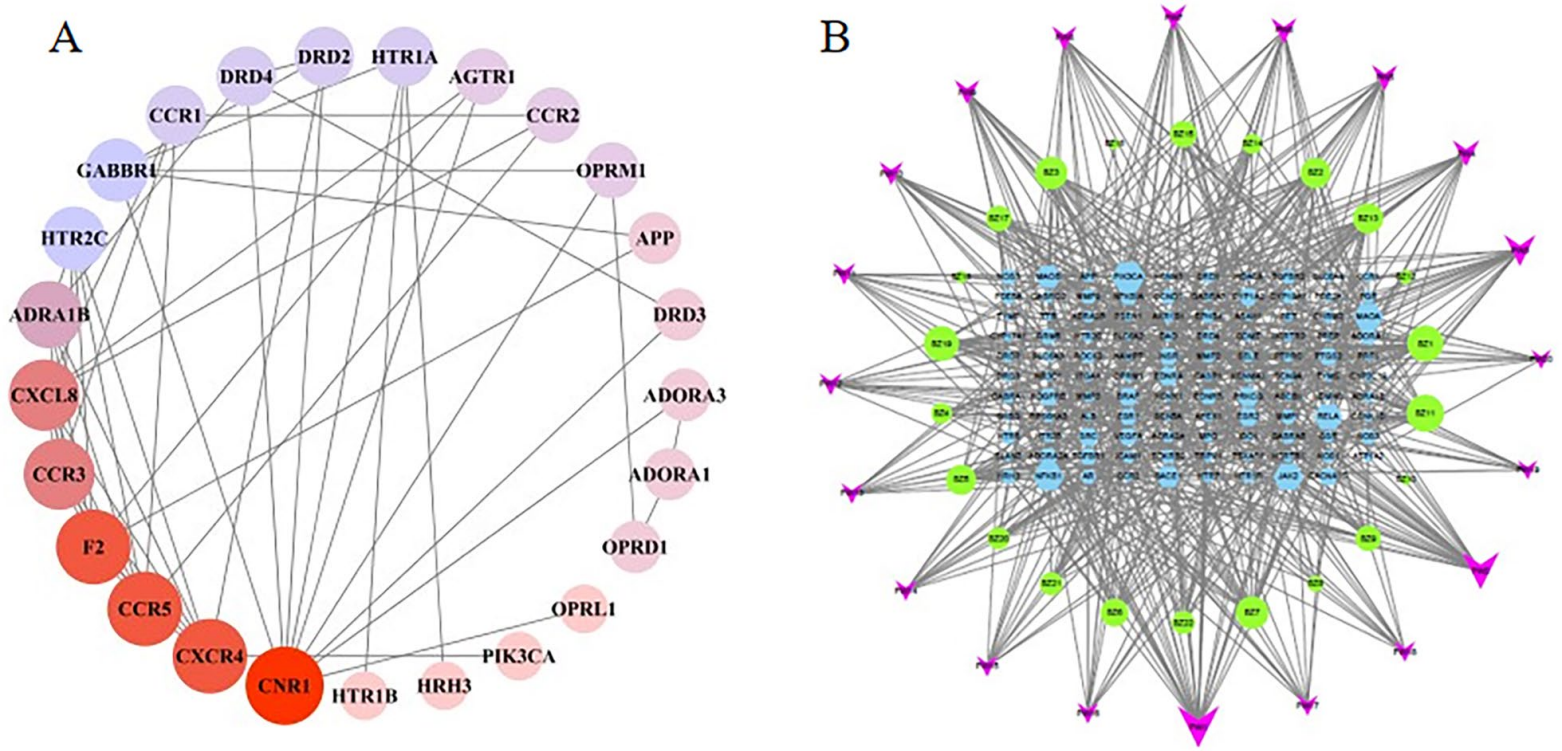
The network of different categories. Protein–protein interaction networks whose nodes are protein (**A**). Compound-target-signaling pathway networks are composed of compound nodes, protein nodes, and signaling pathway nodes (**B**)

In network pharmacology, a network is defined as the linking of targets, compounds, signaling pathways, and other elements in a specific way, forming a network diagram of interactions. Network visualization is about extracting interaction information from linked data and using tools to visualize and analyze [61]. Depending on the kind of nodes, networks can be grouped into 2 main types: single-element networks whose nodes represent the same type of element (such as protein–protein interaction networks) (Fig. 2A) and multi-element networks whose nodes contain multiple types of elements (such as drug-target-disease networks and drug-target-pathway networks) (Fig. 2B). The difference in network properties not only affects the visualization of the final result but also offers the choice of method for network analysis and the topology of the network academic properties [62].

There are two steps in network visualization: (1) the addition of network nodes, the establishment of links between them, and the assignment of attributes to them; (2) the description of the network and the extraction of abundant instruments to characterize the framework feature that visibly stands for the network [61]. Network pharmacology visualization is often achieved through professional software such as Cytoscape. Jian Yang [63] constructed a visual network of eight prescriptions and herbs and analyzed the network to classify the eight prescriptions into five categories, which showed the similitude of the category of the prescription at the herb level. The network analysis is used to capture useful information in the network by extracting targets and drugs to obtain key targets, active compounds, and metabolic pathways. Several approaches are employed for network analysis, typically involving network structure analysis, network function analysis, and network analogy analysis [64].

### The application of network pharmacology in TCM

**Fig. 3:**
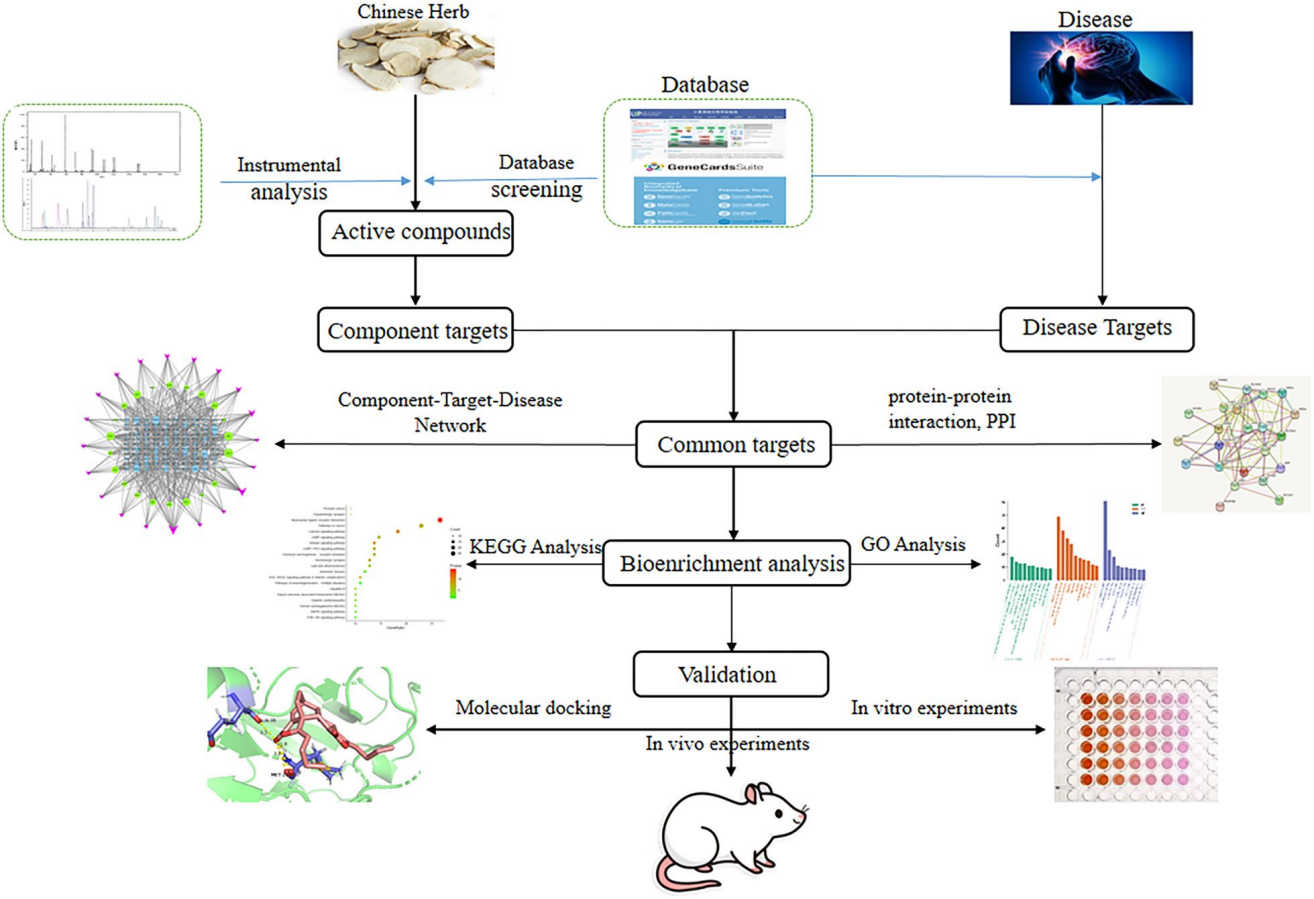
Schematic diagram of the utilization strategy of network pharmacology in TCM research

Network pharmacology research strategies are clear and easy to apply, so they have been used in many directions of TCM research. Network pharmacology offers an opportunity that shifts the research strategy of TCM prescriptions from empirical to evidence-based research. With the utilization of network pharmacology, many aspects of TCM research have been studied and many research results are available [35–37, 65, 66]. The research ideas of the network pharmacology strategy in TCM research are displayed in Fig. 3. This section summarizes the utilization of network pharmacology to TCM in four main areas: elucidating the biological basis of diseases and syndromes, predicting targets for TCM, screening for bioactive substances, and deciphering mechanisms.

#### Elucidating the biological basis of diseases and syndromes

It is challenging to clarify the biological basis of syndromes and diseases because of the complicated philosophy of Chinese medicine and the unclear pathophysiology of diseases. Network pharmacology offers opportunities to systematically reveal the causal mechanisms through biomarkers of disease or syndrome by combing with multi-omics. Network pharmacology is not only dependent on the data collection but also on the algorithmic scores. Algorithms have also been developed for cancer drug sensitivity calculation and cancer biomarker screening. Ali Oskooei et al. [67] have used a new algorithm called network-based bias tree ensembles which are utilized in the calculation of cancer drug sensitivity and the decipherment of agent sensitivity biomarkers. The network-based bias tree ensembles algorithm is more reliable in predicting IC50 drug sensitivity than the unbiased randomized forests algorithms which is only applicable to the targeted membrane receptor pathway. Based on their findings, they proposed that for agents that down-regulate the targeted membrane receptor pathway, the levels of targeted genes before administration are the biomarkers of IC50 drug sensitivity [67].

Network pharmacology combined with multi-omics has been employed to screen the biomarkers of different diseases for clarifying the biological basis of diseases and TCM treatment. Zhang et al. [68] employed a comprehensive strategy based on metabolomics and network pharmacology for the accurate screening of diagnostic biomarkers. They explored the diagnostic biomarkers through systems pharmacology and metabolomics-based techniques and developed a viable pharmacological model. They used this model to identify 23 biomarkers for the treatment of acute ulcerative colitis with *Pulsatilla chinensis* (Bge.) Regel decoction (PD). Fang Sheng-Quan [69] combined network pharmacology with transcriptional profiling approaches to analyze and identify biomarkers for the in vivo treatment of gastric cancer with the Huosu Yangwei Formula. Dong et al. [70] identified 18 potential biomarkers that are associated with insomnia and mood disorders and found that the metabolic markers were altered in mice intervened with *Sedum* L. using metabolomic technique and network pharmacology approaches. Wang et al. [71] investigated the pharmacodynamic components and bioactive mechanisms of *Dendrobium chrysostom* Lindl. in treating precancerous lesions of gastric cancer via network pharmacology. The results suggested a possible therapeutic effect by modulating the HRAS-PI3K-AKT pathway. Acute myeloblastic leukemia is a type of malignant disease, that originates from the malignancy of stem cells, and patients with this acute myeloblastic leukemia have a high risk of death. Yuan et al. used a non-targeted metabolomic technique and network pharmacology-based strategy to decipher its pathogenesis, identifying 75 biomarker targets [72]. Sun et al. explored the common biomarkers of coronary heart disease and hyperlipidemia by integrating lipidomic techniques and network pharmacology [73]. Using network pharmacology and lipidomics strategy, berberine was demonstrated to regulate glycerophospholipid and sphingolipid metabolism for treating hyperlipidemia, and 13 metabolites were discovered as biomarkers of berberine regulation [74].

The advanced detection technology combined with network pharmacology method to explore biomarkers of diseases and symptoms is a new approach to pharmaceutical research. The combined use of network pharmacology and metabolomics is favored by some academics. This approach identified 5 key biomarkers of peonidin against cholestatic liver injury [75], 20 potential biomarkers and 10 major pathways of immune regulation of macrophages by ginseng [76], and 21 biomarkers of type 2 diabetes [77]. The metabolomics analysis identified phosphatidylcholine and phosphatidylethanolamine as biomarkers of Long Chai Fang against duck hepatitis B virus [78]. The research suggested that TNF-*α*, iNOS, MAPK3, eNOS, mTOR, and COX2 are implicated in the immune and inflammatory responses caused by psoriasis [79]. Network pharmacology was utilized to decode the associations between metabolic biomarkers and depression, and 9 metabolite targets correlated with the neurological, immune, and endocrine systems are potential drug targets for the treatment of depression, including PTEN, TP53, HLA-DRB1, bIL1B, TNF, HRAS, MTOR, PIK3CA, and INS [80]. It was demonstrated that Qi-Fu-Yin had a modulating effect on iNOS, a neuroinflammatory biomarker of Alzheimer's disease, as verified by online pharmacological analysis and experiments [81]. Another research has revealed that MMP9, QPCT, and CA1 were potential bioactive markers for patients with nonunion and targets for *Dipsacus asper* Wall.ex Henry [82]. In summary, the network pharmacology approach has been proved useful in identifying active ingredients and elucidating the material basis and mechanisms of biomarkers.

#### Predicting the targets of TCM

The traditional technique for identifying the drug target is to use small molecules as probes, and the biochemical methods study the expression of relevant proteins or the specific binding of small molecules to protein macromolecules to locate the specific binding sites. The identification of agent targets has become a hot study field in Chinese herbal preparations, and drug target discovery is a critical progress in drug research [83]. Network pharmacology is a comprehensive method combining computer-aided algorithms and virtual models to predict multi-target, which is widely employed for the identification of new targets for complicated TCM formulas. Guo et al. [84] used the network-based pharmacological strategy to discovery the major targets for the anti-inflammatory activities of *Lonicera japonica* Thunb (honeysuckle)using the network pharmacology method and validated its effects by the molecular docking and ex vivo verification experiments. The results demonstrated that honeysuckle can significantly down-regulate the levels of AKT and TNF-*α* in RAW264.7 cells. Other scholars have predicted the anti-inflammatory mechanisms of different drugs. The main targets predicted for the anti-inflammatory action of *Lantana camara* L. were IL2, RELA, PRKCA, FOS, and MAPK 14 [85]. Han et al. [86] employed experimental validation and network pharmacology to identify the targets of Curculigoside A in rheumatoid arthritis and osteoporosis. Tao et al. [87] analyzed the molecular targets of the Huashi Baidu formula for the medical Intervention of COVID-19 via integrating molecular docking validation with network pharmacology.

The development of agents for treating cancer is now becoming a major hot topic, and TCM for cancer has received a lot of interest from researchers, with most of the relevant studies already available. However, the traditional methods of mechanism research of TCM for cancer treatment can only reveal limited targets. With the development and promotion of network pharmacology, it is possible to effectively reveal potential targets. Through the application of network pharmacological and in vitro experiments, it was showed that *Astragalus membranaceus* (Fisch.) Bge. var. *mongholicus* (Bge.) Hsiao (Huangqi) promotes apoptosis in colorectal cancer cells by reducing the levels of CXCL10, PTGS2, CXCL8, and CCL2 genes [88]. Besides, it was suggested that AKT1, JUN, CDKN1A, BCL2L1, and NCOA1 are the major targets for Zuojinwan in the therapy of colorectal cancer [89]. Lan et al. [90] explored the main targets of *Rheum palmatum* L. for cancer treatment, and their results indicated that targets such as JUN, CASP3, TNF, MYC, and PTGS2 were involved in the anti-tumor treatment of *Rheum palmatum* L. (Rhubarb). The results from triple-negative breast cancer studies have shown that Curcumin and its nano-formulations down-regulated the expression levels of 10 key genes, including TNF, STAT3, PTGS2, AKT1, EGFR, MMP9, PPARG, NFE2L2, GSK3B, and EP300 [91]. In Qingli Cui's research, 23 targets were revealed for ginseng extracts for treating gastric cancer [92]. Gong et al. [93] successfully predicted the bioactive compounds and main targets of *Codonopsis pilosula* (Franch.) Nannf against Osteosarcoma, with TP53, HSP90AA1, CCND1, and AR playing a significant role in the clinical intervention. The network pharmacology approach is well applied in the prediction of cancer targets for Chinese medicine treatment.

In addition, network pharmacology is used in the treatment of other pathological disorders via Chinese medicine. Specifically, it was applied to the prediction of potential targets for Acteoside therapy [94], identification of targets of *Salvia miltiorrhiza* Bge. against Oral Submucous Fibrosis [95], and investigation of the key target of Rhamnocitrin against Oxaliplatin-induced neuropathic pain [96]. Besides, several researchers have predicted the therapeutic targets for diabetes and diabetic nephropathy, including TNF, TP53, PTGS2, MAPK1, AKT1, IL-6, JUN, and MMP9 [97–100]. Chen et al. [101] utilized a network pharmacology method to identify MAPK1, MMP9, NFKBIA, and other targets that were involved in the treatment of myocardial infarction with Suxiao xintong by dropping pills. Network pharmacology has also been used to explore key targets for the treatment of allergic rhinitis with Tuomin-Zhiti-Decoction [102], the exploration of potential targets for immuno-inflammatory in patients with ankylosing spondylitis [103], and the clarification of targets for Aidi injection for treating pancreatic cancer [104]. Based on network pharmacology, Beihui He et al. revealed that JUN, PTGS2, CDKN1A, and MYC were the targets of Shenqi Pill for treating non-alcoholic steatohepatitis via network pharmacology. In another study, VEGF-C was predicted as an important target of flavonoids from *Citrus* × *limon* (Linnaeus) Osbeck against non-alcoholic steatohepatitis through network pharmacology [105, 106]. Through network pharmacology, it was indicated that the hepatoprotective effects of *Salvia miltiorrhiza* Bge. were tightly correlated with the modulation of intracellular PPAR*α*, CYP1A2, and MMP2 [107].

#### Screening bioactive substances

The lack of clarity of effective substances in Chinese herbal formulas is one of the critical problems causing the bottleneck in the research of Chinese herbal formulas. Hence, there is an urgent need for an integrated approach that can identify multiple bioactive compounds [108]. Network pharmacology provides a simple way to map the potentially effective components into the disease gene networks to find the pharmacodynamic substances. For instance, KiKwangOh et al. [109] employed a network pharmacology approach to identify the bioactive substances associated with diabetes in *Sorghum bicolor* (L.) Moench, ultimately identifying four bioactive substances associated with diabetes. Zhang et al. [110] identified 6 bioactive components of ginger for colon cancer prevention through an integrated network pharmacological approach. Man Kit Cheung and Li Han have studied separately active ingredients of *Andrographis paniculata* (Burm. f.) Nee in esophageal cancer treatment and anti-gastric-cancer effect of herbal Sarcandra glabra (Thunb.) Nakai. The former identified a total of 22 potential therapeutic ingredients for esophageal cancer [111], while the latter identified a total of 6 active ingredients against gastric cancer [112]. In the study of the antidepressant component of *Rehjnannia glutinosa* Libosch., the main active ingredients included melittoside, catalpol, gardoside, genameside C, genipin-1-gentiobioside, and 6-O-p-coumaroyl ajugol [113]. Network pharmacological studies showed that the antibacterial bioactive substances of *Paeonia rockii* (S. G. Haw & Lauener) T. Hong & J. J. Li were flavonoids and phenolic acids [114]. In addition, 12 active ingredients in *Polygonum cuspidatum* Sieb. et Zucc. (Polygonum) was able to relieve asthma symptoms [115]. From the collagen hydrolysates of Andrias davidianus skin, a peptide named GPPGPA was identified through network pharmacology [116].

Different scholars have studied the anti-inflammatory active ingredients of Pudilan Xiaoyan Oral Liquid and Ba-Wei-Long-Zuan Granule. There were 41 potential bioactive ingredients in Pudilan Xiaoyan Oral Liquid [117], and the most relevant active ingredients of Ba-Wei-Long-Zuan Granule for anti-inflammation were coclaurine and hesperidin [118]. It has also been shown that Huangtu Decoction was able to treat colitis with wogonin considered to be the most effective bioactive substance [119]. Wang et al. [120] combined the phytochemical and network pharmacological approaches to identify the bioactive substances and the underlying mechanism of the drug for treating precancerous lesions in the stomach and validated 13 bioactive substances. Besides, 25S-macrostemonoside P was believed to be the bioactive substance of Gualou Xiebai Decoction in alleviating coronary heart disease [121]. Meanwhile, network pharmacology and molecular docking technologies were applied to identify the bioactive substances of *Cuscuta australis* R. Br. (Cuscutae Semen) in the therapy of Osteoporosis [122], revealing the basis of biological activity of Xuanbai Chengqi decoction for treating COVID-19 [123], and discover the pharmacodynamic compounds of Cuscutae Semen-*Morus alba* L. coupled-herbs in treating Oligoasthenozoospermia [124].

The quality of herbs is also an important factor in ensuring efficacy. The introduction of Q-markers has established a value framework for quality control in Chinese medicine [125], and the use of network pharmacology methods to screen herbal quality markers has been accepted by many researchers. Cao et al. predicted the quality markers of *Cistanche deserticola* Y. C. Ma. (Cistanches Herba), and their results preliminarily confirmed echinacoside, acteoside, isoacteoside, cistan oside F, 2′⁃acetylacteoside, cistanoside, and ferulic acid as quality markers for Cistanches Herba [126]. Chen et al. determined 6 flavonoids as the quality markers for *Sophora flavescens* Ait. [127]. Duan et al. screened the quality markers of Wuji Baifeng Pills which include ferulic acid, formononetin, senkyunolide A isoliquiritigenin, and neocryptotanshinone [128]. Network pharmacology coupled with multiple different techniques has been widely utilized to screen Q-markers of Da-Cheng-Qi decoction [129], *Periplocae Cortex* Bge. [130], *Alisma orientale* (Sam.) Juzep. [131], *Trifolium pretense*. L [132], Baizhu dispensing granules [133], and Xinjiang *Cydonia oblonga* Mill. for antiatherosclerosis [134]. Lan et al. [135] analyzed the quality markers of *Rheum palmatum* L. in Fengyin Decoction through fingerprinting and network pharmacology and tentatively identified compounds such as Rhubarb anthraquinone as the potential quality marker. Network pharmacology-metabolomics-PK/PD modeling was used to screen quality markers for the therapy of acute lung injury with Qingzao Jiufei decoction [136], a total of 9 representative components were extracted from the 15 active compounds as the quality markers.

#### Deciphering mechanisms

The clinical medication characteristics of TCM are multiple components, targets, and signaling pathways of action, making it hard to clarify the underlying mechanism with the traditional methods. With the rise and popularity of network pharmacology, it has become an effective approach to utilize network pharmacology to discover the pharmacodynamic substances and active mechanisms of TCM [23]. Deng et al. [137] utilized the network pharmacology strategy to decipher the pharmacodynamic compounds and the molecular mechanisms of the anti-inflammatory and antiviral effects of *Isatis indigotica* Fortune and its mechanism of action. The findings suggested that the indoles, lignans, and flavonoids of *Isatis indigotica* Fortune exert anti-inflammatory and antiviral efficacy through suppressing IL-1*β* and TNF-*α* expression and regulating the immune system and PI3K-Akt signal pathway. Cui et al. elucidated that the anti-inflammatory pharmacological activity of tanshinone I and cryptotanshinone, components of *Salvia miltiorrhiza*, relates to the down-regulation of TLR signaling pathway and the regulation of iNOS synthesis [138]. Zhang et al. [139] investigated the molecular mechanisms of *Salvia miltiorrhiza* for treating diabetic nephropathy by combining the molecular docking and network pharmacology methods. The results of molecular docking demonstrated that tumor necrosis factors, NOS2 and AKT1, the targets of diabetic nephropathy, had higher binding to salvianolic acid B than salvianolic acid A. Other researchers have deciphered the mechanisms of different herbs in the therapy of diabetes and its complications, including *Coptis chinensis* Franch. (Coptidis) [140], Gegen Qinlian decoction [141], Silkworm excrement [142], Qingrekasen granule [143], and Yishen capsules [144]. Guo et al. [145] used a network pharmacology strategy to study the therapeutic mechanisms of TCM formula Zuojin Pill against hepatocellular carcinoma. Both in vitro and in vivo results suggested that Zuojin Pill regulates cell proliferation by modulating the expression of EGFR/MAPK, PI3K/NF-κB, and CCND1, showing significant therapeutic effects in hepatocellular carcinoma.

Several researchers have studied the active mechanism of different herbs for the treatment of the same disease. Although the pharmacological mechanisms of different herbs are not the same, the TCM theory expressed as “the same disease with different treatments” can be elucidated using network pharmacology. The efficacy of Hydroxysafflor yellow A against ischemic stroke is mediated through related to the control of oxidative stress and cellular and vascular renewal [146]. Besides, another experimental validation revealed the pharmacologic action of luteolin on ischemic stroke via the TNF signaling pathway [147]. Cui et al. and Fan et al. investigated the cardioprotective and angiogenesis mechanisms of different herbal medicines for treating myocardial infarction [148].

In addition, network pharmacology has also been carried out to explore the therapeutic mechanisms of many diseases including the treatment of recurrent respiratory infections with improved *Panax ginseng* C. A. Mey (Ginseng)-Schisandra [149], Nauclea officinalis against LPS-induced acute lung injury [150], *Coptis chinensis* treat Kawasaki disease [151], and *Astragalus complanatus* R. Br. anti-aging [152]. The mechanism of action of Shenling Baizhu powder on pyrotinib-induced diarrhea the therapeutic mechanisms of *Astragalus mongholicus* Bunge in non-alcoholic steatohepatitis, and the anti-Hashimoto thyroiditis effect of *Prunella vulgaris* L. were all the result of multiple signaling pathways acting together [153–155]. Besides, *Bos taurus domesticus* Gmelin effectively protected neurovascular units in the brain through HIF-1*α*, PI3K/Akt, and VEGF signaling, thereby affecting the development of ischemic stroke [156]. In the study of arthritis, total flavonoids of *Drynaria fortunei* (Kunze) J. Sm. in the drug intervention of rheumatoid arthritis involved in multiple signaling pathways, particularly closely linked to the inhibition of Th17 differentiation and synovial cell inflammatory responses [157]. The application of *Aconiitum carmichaelii* Debx.-*Cinnamomum cassia* Presl for treating osteoarthritis was largely associated with apoptosis and mitochondrial functional metabolism, which inhibited chondrocyte degeneration by inhibiting the protein and mRNA levels of chondrocyte caspase-3 and promoting the synthesis of ATP [158]. Chen et al. [159] predicted that the underlying mechanisms of Angong Niuhuang Pills for treating neoplastic pneumonia was related to the compound binds to multiple targets which in turn participate in the JAK-STAT and MAPK signaling pathways, thus exerting a therapeutic effect. It was revealed that *Curcuma aromatica* Salisb. significantly improved blood stasis, myocardial infarction, and lipid levels through regulation of the PI3K/AKT/mTOR signaling pathway in rats with coronary artery disease [160]. Network pharmacology and the experimental validation have shown that Polygonum-*Ligustrum lucidum* Ait. acted as a therapeutic agent for acute gouty arthritis by modulating IL-1*β*,TNF-*α*, and IL-6 targets [161]. The above studies showed that the combination of molecular docking, network pharmacology, and experimental validation could elucidate the therapeutic mechanisms of TCM for preventing and treating diseases, which was a simple and practical approach. A various of methods include pharmacokinetics, molecular docking, different omics techniques, as well as pharmacology experiments have been widely employed to decipher the mechanism of action of TCM by integrating with network pharmacology (Table 2).

**Table 2:** Deciphering mechanism of action of TCM using network pharmacology

Disease	TCM	Methods	Effects and Mechanisms	Refs
Type 2 diabetes mellitus	Rhizoma Coptidis	Network pharmacology, Molecular docking, Experimental validation	IL6, VEGFA, and TNF could stably bind with all active compounds of Rhizoma Coptidis and Rhizoma Coptidis could inhibit the expression of IL6 and TNF-*α* and enhance islet cell viability	[140]
Type 2 diabetes mellitus	Silkworm excrement	THE network pharmacology combined with experimental verification	AMPK/PI3K/Akt signaling was an important way for the anti-type 2 diabetic activity of silkworm excrement	[141]
Nephrotic syndrome	Qingrekasen granule	Integrated metabolomics, Network pharmacology	Promoted autophagy and anti-apoptosis through the expression of AKT1, CASP3, BCL2L1 and mTOR, thereby protecting podocytes and maintaining renal tubular function	[142]
Diabetic nephropathy	Yishen capsules	Network pharmacology	The active constituents of Yishen capsules modulated targets or signaling pathways in DN pathogenesis	[143]
Recurrent respiratory infections	Improved Panax ginseng C. A. Mey (Ginseng)-Schisandra	Network pharmacology	Modified Ginseng-Schisandra Decoction was able to treat RRTI primarily through acting in the signal transduction of some key nodes of cancer pathway and TNF pathway	[149]
Hepatocellular carcinoma	Zuojin pill	Network pharmacology	The compound-target network included 32 compounds and 86 targets, whereas the target-pathway network included 70 proteins and 75 pathways	[162]
Pyrotinib-induced diarrhea	Shenling Baizhu powder	Gut microbiota, Metabonomics, Network pharmacology	The regulation of inflammatory bowel disease, IL-17 signaling pathway, pathogenic Escherichia coli infection and cAMP signaling pathway, were involved in the therapeutic effect of Shenling Baizhu powder against pyrotinib-induced diarrhea	[163]
Blood-heat and blood stasis syndrome	Moutan Cortex	Pharmacokinetics, Network pharmacology, Molecular docking	F2, F10, F7, PLAU, MAPK14, MAPK10, AKT1, and NOS3 were screened as targets regulated by raw Moutan Cortex for the treatment of blood-heat and blood stasis syndrome	[164]
Alzheimer's disease	Chuanxiong Renshen decoction	Network pharmacology, UPLC-Q–TOF–MS, Molecular docking	The downregulation of CASP3 and EGFR were involved in the therapeutic effect of Chuanxiong Renshen decoction against Alzheimer's disease	[165]
Hepatic steatosis	Hawthorn or semen cassiae	Network pharmacology	Hawthorn/semen cassiae treatment lowered expression of PPAR-*γ* and GRP78, thereby ameliorating ER stress and hepatic steatosis	[166]

## Network pharmacology as the opportunity for the personalized precise medication of TCM

**Fig. 4:**
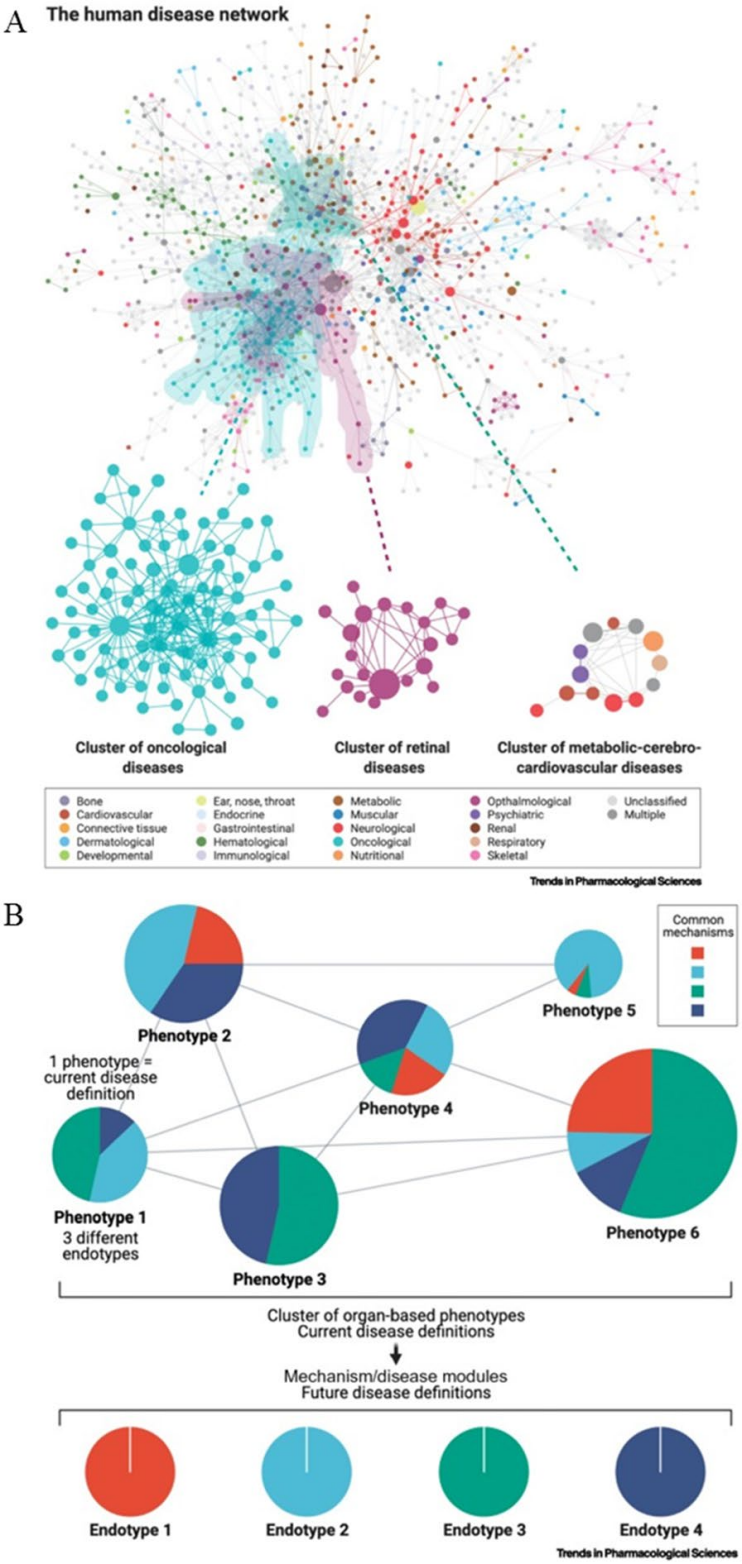
Human disease networks and endotyping disease clusters. Human disease networks (**A**); endotyping disease clusters (**B**). Reproduced from Ref. [24]

Diseases are abnormal processes of the bodily activity triggered by a disruption in the self-regulation of body under the influence of certain factors, resulting in an associated disease phenotype. Because of the complicated network of human diseases and the incomplete understanding of the causes of diseases, diseases are always clarified by symptoms manifesting in organs. Thus, diseases are mainly treated by curing the symptoms, but not the cause. Currently, scientists have started to realize that the development of a disease can be described as the dysregulation of one or more interacting network processes, as described by Nogales [24] (Fig. 4A). Network pharmacology is a research strategy that focuses on “network targets”. As a result, it is a reasonable approach to characterize both the manifestation and the root cause of diseases by taking advantage of integrated and systematic biology approaches, such as epigenomics, transcriptomics, proteomics, and metabolomics [167, 168]. Disease phenotypes should be categorized by the dysregulation of biological networks rather than mechanistically unrelated proteins (Fig. 4B). Network pharmacology has been a promising method for advancing drug discovery and illuminating the underlying mechanism of multi-targeted compounds [169–171]. It presents diseases as perturbations of intertwined molecular networks and characterizes the therapeutic mechanisms of drugs through the network topology. TCM exerts its therapeutic effect through regulating biological networks, and the potential mechanism of TCM can be explained by using network pharmacology [25]. With the rapid growth of systematic biology and bioinformatic technologies, the network pharmacology is developing rapidly. Network pharmacology provides an opportunity for precisely treating diseases by clarifying both the symptoms and causal mechanisms, enabling personalized precise medication of TCM. The “Evidence-based Precision Medicine in TCM” integrates the evidence-based theory of TCM diseases and the integrated biological data research knowledge network uses the evidence as a benchmark for the comprehensive classification of patients and provides a more precise treatment under the guidance of evidence-based medicine [172]. With the progress of TCM syndrome, the evidence can be initially classified by modern means based on gene expression, which can relatively reduce the dialectical reliance on TCM practitioners. The network pharmacology can decipher the corresponding biomarkers of the syndrome and the mechanisms of drug-drug interactions, providing plenty of effective evidence which can then be used in large-scale clinical trials and employed to verify the efficacy. This will enable the integration of dialectical thinking and evidence-based precision medicine in TCM, realizing personalized precise medication of TCM [83]. Network pharmacology opens up opportunities for the personalized and precise use of TCM by establishing a link between TCM and biological networks at the molecular level. It is essential to explore how network pharmacology can be more scientifically integrated with the theory of Chinese medicine to promote the personalized precise medication of TCM.

## Perspective

With its complex chemical composition and the ill-defined mechanisms of action, TCM has always been treated as an empirically based medicine, but the effectiveness of TCM is undeniable. The conventional “one target-one drug-one disease” research paradigm does not apply to TCM research, hence hindering the conduction of evidence-based study of TCM. Network pharmacology is undoubtedly an excellent adjunct to TCM research, because it connects the old idea of TCM and modern scientific practices. Herein, we summary the utilization of network pharmacology strategy in understanding the formulation of herbs, the therapeutic mechanisms of herbal medicines for treating diseases, the exploration of the potential targets, and identifying the pharmacodynamic material basis of herbal medicines for treating diseases. The hope is a major change in drug development and therapy which follows multiple components, multiple targeted, and multiple pathway manners. With the aging of the population in the East Asian TCM culture circle, the number of patients with multiple chronic diseases is increasing. In the course of long-term medication of TCM, the traditional medication scheme not only is ineluctable to cause the waste of drug resources, but also brings about unsatisfactory treatment effect treatment effects and irreversible damage to the body. Through the utilization of network pharmacology integrating systems biology, bioinformatics, and computer science, accurate prediction of individual complex pathological pathways and personalized prescription medication will be greatly developed. Network pharmacology is a bright guiding light on the way to explore the personalized precise medication of TCM.

Notably, several factors may limit the utilization of network pharmacology, such as the selection of databases and algorithms, the unstable quality of the research results, and the lack of standardization. The integrity of the database data and the rationality and credibility of the algorithms determine the verifiability and trustworthiness of the network pharmacology results. Among the various databases and algorithms, it is not at all clear which explains the mechanisms of drug combination well. For example, the selection of active compounds in herb medicines is often based on indicators of oral bioavailability and drug-like properties which may lack a solid foundation. Besides, the most commonly discussed disadvantage of this method is the reliability of the data interpretation, which depends highly on the database coverage. Fortunately, the development of *Network Pharmacology Evaluation Method Guidance* is a critical step for advancing the development of network pharmacology, which will help to promote the use of the new pharmaceutical research model in a more standardized and rigorous manner and stimulate the development of the new frontiers in the network pharmacology in TCM research [27, 173]. Additionally, the inability of network pharmacology to map complex interactions simultaneously limits the utility in clarifying the relationship among the compounds, gut microbiota, and the host. Finally, most of the network pharmacology methods only construct interaction networks between compounds and targets, losing concentration-related information. Thus, the screened components using network pharmacology may not be the best-acting compounds in herbal medicines because the therapeutic effects cannot be verified. The above-mentioned issues are the main problems and obstacles in the development of network pharmacy. In the era of rapid advances in information technology, there is an unstoppable trend toward the utilization of network pharmacology for the comprehensive and systematic research of TCM. In the future, the network pharmacology has to be constantly improved to ensure its sustainable development and application, for example, combining with other methods and techniques, enriching the content of the various databases, and updating the reliability. In addition, to make the results more convincing and representative, general standards should be set for the network pharmacology analysis methods. Network analysis methods should be improved to make it commonplace. It will also be important to quantify the active ingredient to determine whether it has reached the pharmacodynamic concentration. To prove its credibility, the network pharmacology results should be effectively combined with pharmacology, pharmacokinetics, toxicology, and pharmacodynamic related experiments. Collectively, network pharmacology offers a new opportunity for precisely treating both the manifestation and the root cause of disease.

## Data Availability

Not applicable.

## Abbreviations

AKT: Protein kinase B
AMPK: Adenosine 5’-monophosphate (AMP)-activated protein kinase
AR: Androgen Receptor
ATP: Adenosine 5’-triphosphate
BCL2L1: B-cell lymphoma-2 like 1
BIL1B: Interleukin-1 beta
CA1: Carbonic anhydrase 1
CAMP: Cyclic Adenosine 5’-monophosphate
CASP3: Caspase-3
CCL2: Chemokine ligand 2
CCND1: Cyclin D1
CDKN1A: Cyclin dependent kinase inhibitor 1A
CMAUP: Collective Molecular Activities of Useful Plants
CNKI: Chinese National Knowledge Infrastructure
COVID-19: Corona Virus Disease 2019
COX2: Cyclooxygenase-2
CXCL10: C-X-C motif chemokine ligand 10
CXCL8: C-X-C motif chemokine ligand 8
CYP1A2: Cytochrome P450 family 1 subfamily A member 2
DAVID: The Database for Annotation Visualization and Integrated Discovery
DisGeNET: Generate gene-disease networks
EGFR: Epithelial growth factor receptor
ENOS: Endothelial nitric oxide synthase
EP300: E1A binding protein p300
ER: Estrogen receptor
ETCM: The Encyclopedia of Traditional Chinese Medicine
F10: Coagulation factor X
F2: Coagulation factor II
F7: Coagulation factor VII
FOS: Fos proto-oncogene
GRP78: Glucose regulated protein 78 kD
GSK3B: Glycogen synthase kinase 3β
HERB: A high-throughput experiment and reference-guided database of traditional Chinese medicine
HIF-1α: Hypoxia-inducible factor-1α
HIT: Herbal Ingredients’ Targets Database
HLA-DRB1: Major histocompatibility complex, class II, DR beta 1
HRAS: HRas proto-oncogene
HSP90AA1: Heat shock protein HSP 90-alpha
IL-17: Interleukin 17
IL-2: Interleukin 2
IL-6: Interleukin 6
INOS: Nitric oxide synthase
INS: Insulin
JAK: Janus kinase
JUN: Jun proto-oncogene
KEGG: Kyoto Encyclopedia of Genes and Genomes
MAPK: Mitogen-activated protein kinase
MMP: Matrix metalloproteinase
MTOR: Mammalian target of rapamycin
MYC: MYC proto-oncogene
NCOA1: Nuclear Receptor Coactivator 1
NFE2L2: Nuclear factor, erythroid 2 like 2
NFKBIA: NFKB inhibitor alpha
NF-κB: Nuclear factor-kappa B
NOS: Nitric Oxide Synthase
OMIM: Online Mendelian Inheritance in Man
PI3K: Phosphatidylinositide 3-kinases
PIK3CA: Phosphatidylinositol-4,5-bisphosphate 3-kinase catalytic subunit alpha
PLAU: Urokinase plasminogen activator
PPARG: Peroxisome proliferator activated receptor gamma
PPARα: Peroxisome proliferator activated receptor α
PPAR-γ: Peroxisome proliferator activated receptor γ
PRKCA: Protein kinase C alpha
PTEN: Phosphatase and tensin homolog deleted on chromosome ten
PTGS2: Prostaglandin-endoperoxide synthase 2
QPCT: Glutaminyl-peptide cyclotransferase
RAW264.7cells: Mouse Mononuclear Macrophage Leukemia Cells
RELA: RELA proto-oncogene
RRTI: Recurrent respiratory tract infection
STAT: Signal transducer and activator of transcription
STAT3: Signal transducer and activator of transcription 3
TCM: Traditional Chinese Medicine
TCMID: Traditional Chinese Medicines Integrated Database
TCMSP: Traditional Chinese Medicine Systems Pharmacology Database and Analysis Platform
TLR: Toll-like Receptor
TNF: Tumor necrosis factor
TP53: Tumor protein p53
TTD: Therapeutic Target Database
UK: United Kingdom
VEGF: Vascular endothelial growth factor
WOS: Web of Science
YATCM: Yet another Traditional Chinese Medicine database
